# Diagnostic accuracy of the Panbio COVID-19 antigen rapid test device for SARS-CoV-2 detection in Kenya, 2021: A field evaluation

**DOI:** 10.1371/journal.pone.0277657

**Published:** 2023-01-25

**Authors:** Jack Karuga Irungu, Peninah Munyua, Caroline Ochieng, Bonventure Juma, Patrick Amoth, Francis Kuria, John Kiiru, Lyndah Makayotto, Ahmed Abade, Marc Bulterys, Elizabeth Hunsperger, Gideon O. Emukule, Clayton Onyango, Taraz Samandari, Beth A. Tippett Barr, Victor Akelo, Herman Weyenga, Patrick K. Munywoki, Godfrey Bigogo, Nancy A. Otieno, Jackton Azenga Kisivuli, Edwin Ochieng, Rufus Nyaga, Noah Hull, Amy Herman-Roloff, Rashid Aman

**Affiliations:** 1 Field Epidemiology and Laboratory Training Program, Ministry of Health, Nairobi, Kenya; 2 U.S. Centers for Disease Control and Prevention, Nairobi, Kenya; 3 Kenya Medical Research Institute (KEMRI), Center for Global Health Research, Nairobi, Kenya; 4 Ministry of Health, Nairobi, Kenya; 5 Directorate of Health, Kenya Prisons Service, Nairobi, Kenya; 6 Association of Public Health Laboratories (APHL), Nairobi, Kenya; University of Cape Town Faculty of Health Sciences, SOUTH AFRICA

## Abstract

**Background:**

Accurate and timely diagnosis is essential in limiting the spread of SARS-CoV-2 infection. The reference standard, rRT-PCR, requires specialized laboratories, costly reagents, and a long turnaround time. Antigen RDTs provide a feasible alternative to rRT-PCR since they are quick, relatively inexpensive, and do not require a laboratory. The WHO requires that Ag RDTs have a sensitivity ≥80% and specificity ≥97%.

**Methods:**

This evaluation was conducted at 11 health facilities in Kenya between March and July 2021. We enrolled persons of any age with respiratory symptoms and asymptomatic contacts of confirmed COVID-19 cases. We collected demographic and clinical information and two nasopharyngeal specimens from each participant for Ag RDT testing and rRT-PCR. We calculated the diagnostic performance of the Panbio^™^ Ag RDT against the US Centers for Disease Control and Prevention’s (CDC) rRT-PCR test.

**Results:**

We evaluated the Ag RDT in 2,245 individuals where 551 (24.5%, 95% CI: 22.8–26.3%) tested positive by rRT-PCR. Overall sensitivity of the Ag RDT was 46.6% (95% CI: 42.4–50.9%), specificity 98.5% (95% CI: 97.8–99.0%), PPV 90.8% (95% CI: 86.8–93.9%) and NPV 85.0% (95% CI: 83.4–86.6%). Among symptomatic individuals, sensitivity was 60.6% (95% CI: 54.3–66.7%) and specificity was 98.1% (95% CI: 96.7–99.0%). Among asymptomatic individuals, sensitivity was 34.7% (95% CI 29.3–40.4%) and specificity was 98.7% (95% CI: 97.8–99.3%). In persons with onset of symptoms <5 days (594/876, 67.8%), sensitivity was 67.1% (95% CI: 59.2–74.3%), and 53.3% (95% CI: 40.0–66.3%) among those with onset of symptoms >7 days (157/876, 17.9%). The highest sensitivity was 87.0% (95% CI: 80.9–91.8%) in symptomatic individuals with cycle threshold (Ct) values ≤30.

**Conclusion:**

The overall sensitivity and NPV of the Panbio^™^ Ag RDT were much lower than expected. The specificity of the Ag RDT was high and satisfactory; therefore, a positive result may not require confirmation by rRT-PCR. The kit may be useful as a rapid screening tool only for symptomatic patients in high-risk settings with limited access to rRT-PCR. A negative result should be interpreted based on clinical and epidemiological information and may require retesting by rRT-PCR.

## Introduction

The COVID-19 pandemic has had major negative socioeconomic effects [1]. Prompt diagnosis of SARS-CoV-2 infection is critical in controlling the pandemic [2]. Real-time reverse transcription-polymerase chain reaction (rRT-PCR) remains the reference standard for the diagnosis of SARS-CoV-2 infection [3]. However, rRT-PCR has limitations, including the need for specialized laboratories, equipment, and staff, longer turnaround time (TAT) for clinical decision making and prevention measures, high cost, and erratic supply of reagents to conduct SARS-CoV-2 rRT-PCR due to increased demand [3–6]. These limitations have increased the demand for more rapid, cheaper, and easy to perform testing methods [7].

Prompt identification of infected individuals is key in initiation of proper management of cases and the implementation of interventions to limit transmission [2]. Antigen rapid diagnostic tests (Ag RDTs) increase patients’ access to diagnosis of SARS-CoV-2 infection as they can be used as point of care tests thereby providing results more prompty. The World Health Organization (WHO) recommends Ag RDTs with a minimum sensitivity and specificity ≥80% and ≥97%, respectively where rRT-PCR is unavailable or is associated with long TAT [8].

The performance of Ag RDTs depends on the presence of symptoms, duration since onset, viral load and the disease prevalence [9]. At the time of study conception, Panbio^™^ Ag RDT was one of two rapid tests approved for SARS-CoV-2 testing in our setup and to the best of our knowledge had not been evaluated. We evaluated the performance of the Panbio^™^ Ag RDT compared to the reference standard rRT-PCR. The Panbio^™^ Ag RDT is authorized in Kenya and received an emergency use license (EUL) from WHO [10]. We also assessed the operational characteristics of the Panbio^™^ Ag RDT in order to identify any user challenges the in the field setting.

## Methods

We conducted a prospective cross-sectional evaluation of the Panbio^™^ Ag RDT in 11 sites in Kenya between March and July 2021. The sites were located in Nairobi County (Nairobi remand Prison, Langata Women’s Prison, and Tabitha level 2 Clinic), Kiambu County (Kihara Sub-county Hospital), Nakuru County (Nakuru Prison), Nyeri County (Nyeri level 5 Hospital and Nyeri Prison), Kisumu County (Kisumu Prison-Kodiaga), Mombasa County (Coast General Teaching and Referral Hospital) and Siaya County (Siaya County Referral Hospital and St. Elizabeth Mission Hospital). The protocol was reviewed and approved by the Kenya Medical Research Institute’s (KEMRI) scientific ethical review unit (SERU). The US Centers for Disease Control and Prevention (CDC) institutional review board provided non-research determination approval. Further administrative approvals were obtained from the Ministry of Health Kenya, the National Commission for Science, Technology, and Innovation (NACOSTI) Kenya, the Kenya Prisons Service, and the county governments of Nakuru, Nyeri, Mombasa, Kiambu, and Siaya. Written informed consent was obtained for all enrolled participants. Written parental informed consent and assent were obtained for participants aged <18 years. Individuals who declined to participate, including prisoners were offered routine COVID-19 services as per national guidelines.

### Clinical case definition for enrollment

We enrolled persons of any age who presented to our study sites with respiratory symptoms (symptomatic participants) that met any of three case definitions, including acute respiratory infection (ARI,) defined as cough or difficulty in breathing or sore throat or coryza, with the onset of symptoms <2 weeks; severe acute respiratory infection (SARI) defined as fever or temperature ≥38°C and cough, with onset of symptoms <10 days and requiring hospitalization; or influenza-like illness (ILI) defined as temperature ≥38°C and cough, with onset of symptoms <10 days. Body temperature measurement was done using infrared thermometers placed 3 to 5 centimeters from the temple. We also enrolled asymptomatic close contacts of individuals with confirmed SARS-CoV-2 infection exposed between two days before and 14 days after symptoms onset or confirmation of infection. Individuals exposed to persons with confirmed SARS-CoV-2 infection in the following setups were considered close contacts in the following scenarios: working in close proximity or sharing the same environment; face-to-face contact within 1 meter for more than 15 minutes; traveling together in any kind of conveyance; living in the same household; healthcare-associated exposure, including providing direct care for COVID-19 patients, visiting patients or staying in the same close environment.

### Data and specimen collection

Individuals who met the case definition and consented were enrolled and assigned unique identifiers. We used pretested questionnaires on Android^®^ smartphones running CommCare^®^ Application to collect demographic and clinical information. Data was stored in a secured cloud server. Clinical information collected included the range and duration of respiratory symptoms, presence of comorbidities, and reported history of exposure to confirmed COVID-19. Two nasopharyngeal swabs were collected by a surveillance officer (nurse, clinical officer, or laboratory technologist) trained to collect respiratory specimens from each of the nostrils a few seconds apart. The first specimen was collected using the swab provided in the Panbio^™^ Ag RDT kit. The second specimen was collected using a polyester-tipped aluminum shafted swab and immediately placed in standard viral transport media prepared in the testing laboratory. The transport media was stored at +2 to +8°C before specimen collection and during shipment to the CDC-supported KEMRI laboratories in Nairobi or Kisumu for rRT-PCR testing. Aliquots of the specimens were then archived at -80°C.

### Rapid SARS-CoV-2 antigen testing

Rapid testing was conducted using the Panbio^™^ Ag RDT (Abbott Rapid Diagnostics, US. Ref. 41FK10) per manufacturer instructions [11] by surveillance officers. The Panbio^™^ Ag RDT is a lateral flow immunoassay that detects the nucleocapsid (N) protein in nasal and nasopharyngeal specimens. Results were read and documented independently by two surveillance officers within 15 to 20 minutes. Positive, negative, and invalid results were interpreted according to the manufacturer’s recommendations. Each reader documented the test results in the electronic questionnaire independently. In the event the two readers documented different results, the final result was indicated as indeterminate.

### Assessing the operational characteristics of the Panbio^™^ Ag RDT kit

We assessed operational characteristics of the Panbio^™^ Ag RDT in the field setting including clarity of kit instructions, technical complexity or ease of use, and the ease of interpretation of results via a standardized questionnaire administered to users on site.

### rRT-PCR testing in the laboratory

According to the manufacturer’s instructions, total nucleic acid material was extracted from 200μL of the nasopharyngeal specimen using either the Ambion MagMax Total RNA isolation kit (Thermo Fisher Scientific, Greenville, North Carolina, USA) performed on a semi-automated KingFisher flex machine (Thermo Fisher Scientific) or Standard M Spin-X Viral RNA Extraction Kit (SD biosensor) according to the manufacturer’s instructions. We then eluted 60uL of total RNA from the extracted sample. The SARS-CoV-2 rRT-PCR was performed using the TaqPath 1-step Multiplex rRT-PCR master mix (Thermo Fisher Scientific) or using the GoTaq^®^ Probe 1-Step RT-qPCR System (Promega A6120). Both systems are compatible with the rRT-PCR CDC IDT kit. Each of the 15μL reaction volumes contained 5μL of master mix, 8.5μL of nuclease-free water, and 1.5μL of CDC-IDT primer/probe mix for each of the N1, N2, and RNP3 genes separately. The eluate was then amplified using QuantStudio^™^ 5 Real-Time PCR System, 0.1ml block (Thermo Fisher Scientific), and analyses were done using QuantStudio 3 and 5 Real-Time PCR System Software version 1.5.1 (Thermo Fisher Scientific). SARS-CoV-2 assay targets, N1 and N2 (N1 and N2) were run simultaneously with the human ribonuclease P gene (RNP) control to monitor the quality of the nucleic acid extraction, specimen quality, and presence of reaction inhibitors for assay performance. The positive, negative, and human specimen controls were included in all assays.

When all controls exhibited expected performance, a positive result for SARS-CoV-2 was considered, if all assay amplification curves crossed the threshold line within Ct <40. If SARS-CoV-2 (N1 and N2) assay results were negative, then the test result was reported as negative. If only one target (N1 and N2) were positive, then it was designated as inconclusive, and retesting was conducted. If upon repeating the test, the results remain inconclusive, then the final result was reported as inconclusive. Results were relayed back to the specific sites for patient management.

### Sample size calculation

To evaluate the diagnostic performance of the Panbio^™^ Ag RDT device, we assumed a prevalence of 10% among symptomatic individuals and 5% in asymptomatic contacts of laboratory-confirmed persons based on current SARS CoV 2 local surveillance data. We assumed a sensitivity of 90% for the Panbio^™^ Ag RDT with 7.5% margin of error, and after adjusting by 10% for other concerns we anticipated enrolling 770 symptomatic patients and 1,540 close contacts of confirmed cases.

### Statistical analysis

Statistical analysis was conducted using Stata version 15.1 (College Station, Texas). Measures of central tendency and dispersion were calculated for continuous variables such as age, days since onset of symptoms, temperature, and cycle threshold (Ct) values. We calculated frequencies and proportions for categorical variables such as age group, occupation, Ct value cut-offs and the operational characteristics of the Ag RDT. The 95% confidence intervals for categorical variables were calculated using the normal approximation method (Wald’s test). We compared proportions between the symptomatic and asymptomatic groups by calculating their Z scores. P-values <0.05 were considered statistically significant.

We calculated the sensitivity, specificity, and positive and negative predictive values for the Panbio^™^ Ag RDT against rRT-PCR as the reference standard. Stratified analysis of performance was done by age, day’s post-onset of symptoms, and Ct values. The measure of agreement between Ag RDT and rRT-PCR results was evaluated using Cohen’s Kappa statistic. We interpreted the strength of agreement as slight agreement (κ, 0–0.2), fair (κ, 0.21–0.4), moderate (κ, 0.41–0.6), substantial (κ, 0.61–0.8), and almost perfect (κ, 0.81–1.0) as described by Landis *et al*. [12].

## Results

### Enrolled participant characteristics

We enrolled 2,279 participants, where 38.9% had at least one respiratory symptom. The median age was 31.0 years (range <1 month– 103 years), and over half (60.9%) were male (Table 1). The median duration post-onset of symptoms among symptomatic participants was three days, with the majority (67.8%) presenting within 5 days.

**Table 1:** Clinical and demographic characteristics of participants in the field evaluation of the Panbio^™^ Ag RDT in Kenya, 2021.

Variable	Symptomatic (886) n (%)	Asymptomatic (1,393) n (%)	Total (2,279) n (%)	*P-*value
**Sex**				
Male	510 (57.6)	877 (63.0)	1387 (60.9)	0.01
Female	376 (42.4)	516 (37.0)	892 (39.1)	0.01
**Symptoms**				
Cough	604 (68.4)	-	-	-
Fever	294 (33.2)	-	-	-
Runny nose	266 (30.1)	-	-	-
Headache	231 (26.2)	-	-	-
Sore throat	197 (22.2)	-	-	-
Shortness of breath	195 (22.0)	-	-	-
**Duration since onset**				
<5 days	601 (67.8)	-	-	
5-≤7 days	126 (14.2)	-	-	
7–14 days	159 (18.0)	-	-	
≤7 days	814 (91.9)	-	-	
>7–14 days	72 (8.1)	-	-	
**Comorbidities and underlying conditions**				
Cardiovascular disease, including hypertension	87 (9.8)	13 (0.9)	100 (4.4)	<0.001
Immunodeficiency, including HIV	31 (3.5)	25 (1.8)	56 (2.5)	0.011
Diabetes	18 (2.0)	6 (0.4)	24 (1.1)	<0.001
Other conditions	43 (4.7)	34 (2.3)	77 (3.2)	0.002
No known comorbidities	720 (81.1)	1266 (90.4)	1986 (86.8)	<0.001
**Other risk factors**				
Smoking (current or former smoker)	11 (1.2)	53 (3.8)	64 (2.8)	<0.001
**Occupational settings**				
Inmates and prison warders	265 (29.8)	1034 (73.9)	1299 (56.8)	<0.001
Health care workers	72 (8.1)	109 (7.8)	181 (7.9)	0.942
Learning institutions	103 (11.6)	64 (4.6)	167 (7.3)	0.123
Preschool	117 (13.2)	21 (1.5)	138 (6)	0.945
Other employment	287 (32.3)	171 (12.2)	458 (20.1)	<0.001
Missing	44 (5)	1 (0.1)	45 (2)	-

n, number of; HIV, human immunodeficiency virus.

### Laboratory findings

#### Panbio^™^ Ag RDT test results.

**Fig 1:**
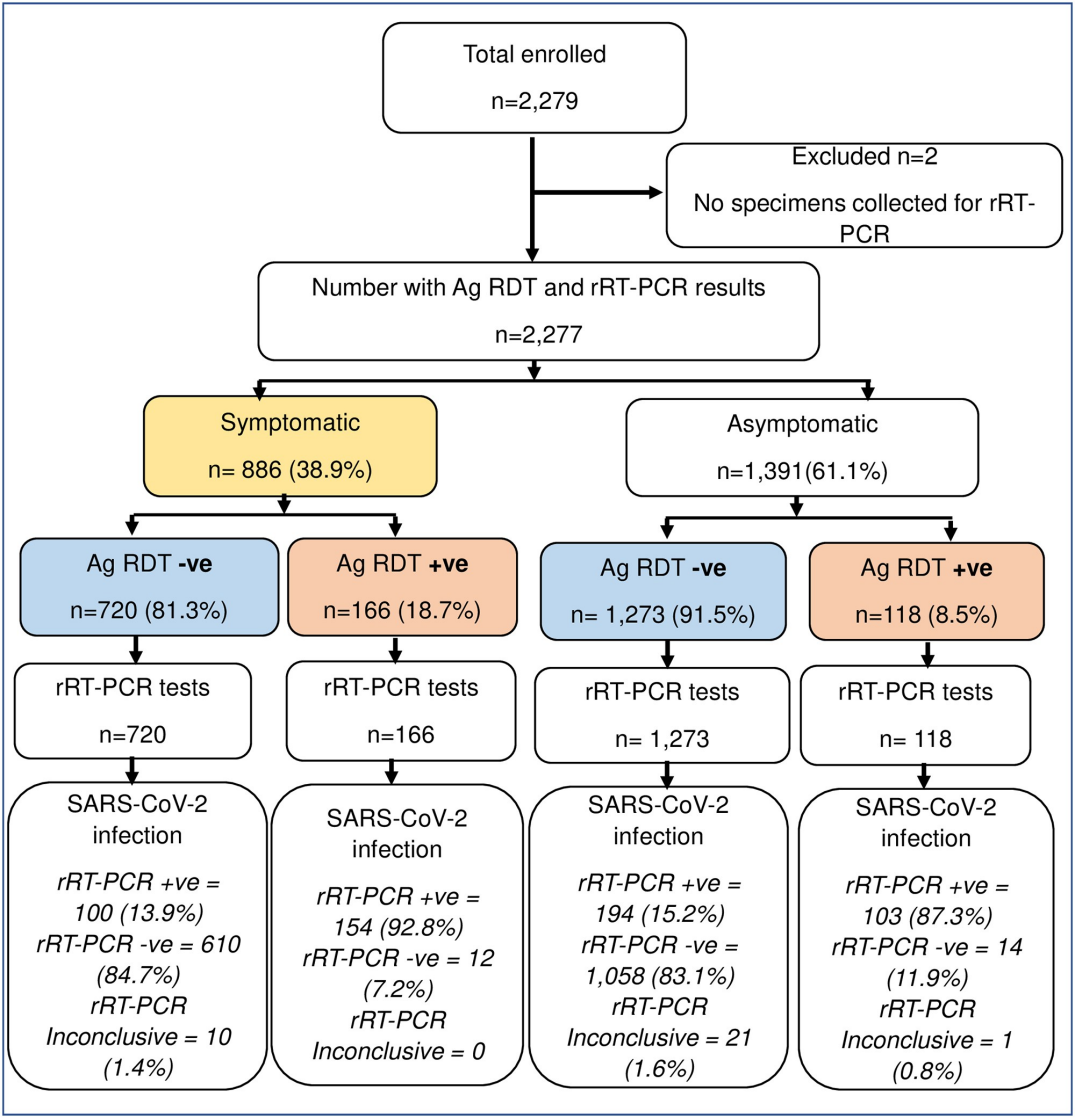
Flow diagram of enrolment of study participants and distribution by clinical status and test results in the field evaluation of the Panbio^™^ Ag RDT in Kenya, 2021. Ag RDT, +ve, +ve, positive; -ve negative; antigen rapid diagnostic test; rRT-PCR–real time reverse transcriptase polymenrase chain reaction.

Among the 2,277 tested using the Panbio^™^ Ag RDT device, 12.4% were positive (Fig 1). There were no invalid or indeterminate Ag RDT results. The positivity was significantly higher among symptomatic individuals (18.7%) compared with the asymptomatic (8.4%,) individuals.

#### rRT PCR test results

**Fig 2:**
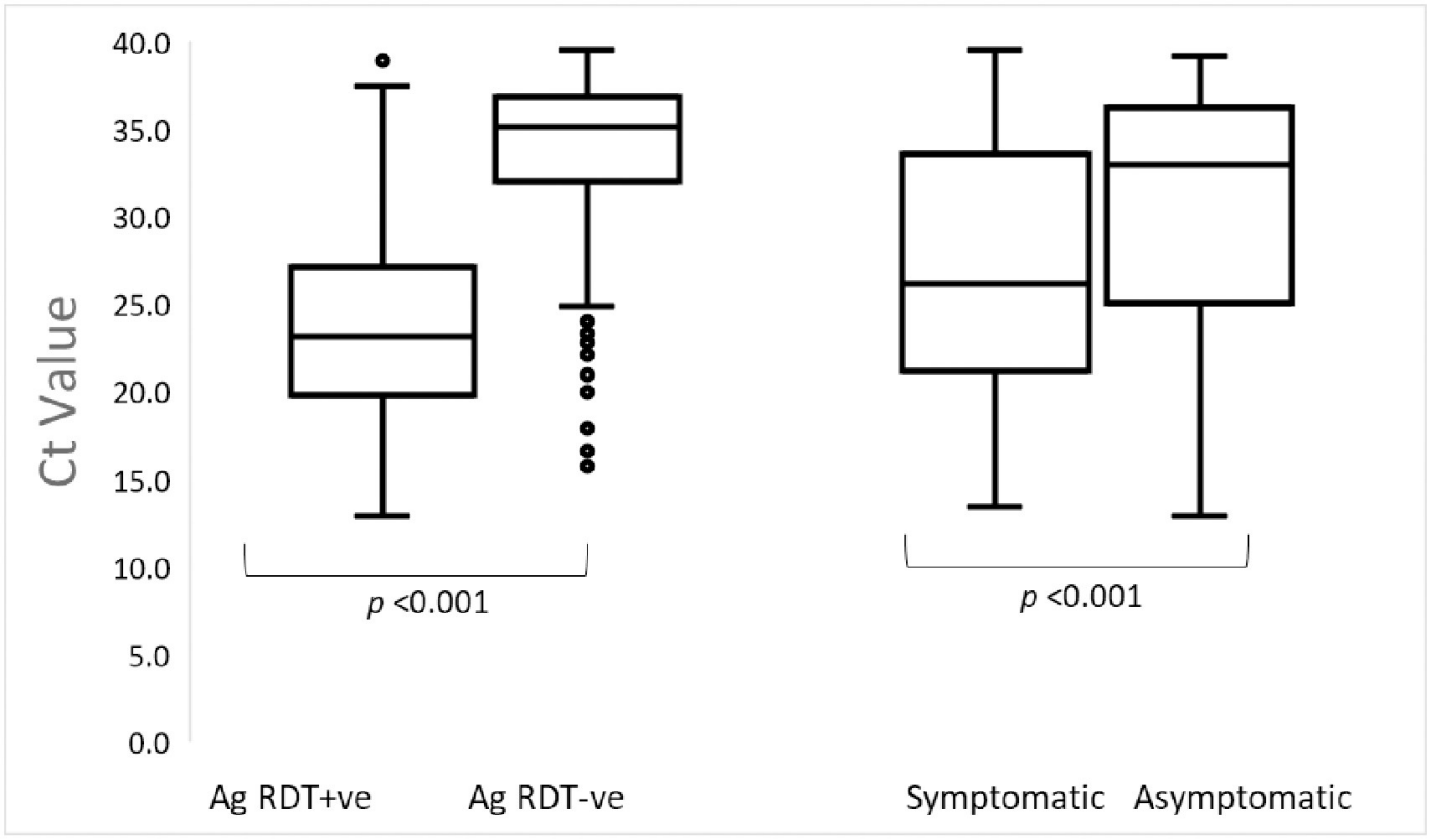
Distribution of Cycle threshold (Ct) values for rRT-PCR positive samples by Ag RDT result and by presence/absence of symptoms, Kenya 2021 (n = 551). Ag RDT, antigen rapid diagnostic test; Ct, cycle threshold; n, number of; rRT-PCR–real time reverse transcriptase polymenrase chain reaction.

We tested 2,277 specimens by rRT-PCR, 24.2% tested positive and 1.4% were inconclusive (Fig 1). The overall median Ct value of the 551 rRT-PCR positive results was 30.2 (Interquartile range (IQR) 22.9–35.6). Symptomatic individuals had a significantly lower median Ct value of 26.2 (IQR 21.1–33.6) compared to 33.0 (IQR 25.0–36.3) among asymptomatic individuals (*p* <0.001). The median Ct value was significantly lower among those with Ag RDT positive results (23.2, IQR 19.8–27.1) compared to negative test results (35.1, IQR 32.0–36.9) (*p* <0.001) (Fig 2).

### Performance of Panbio^™^ Ag RDT

We reviewed 2,245 paired Ag RDT/rRT-PCR records for the performance analysis. The overall sensitivity of the Panbio^™^ Ag RDT was 46.6% (95% CI: 42.4–50.9%) and specificity was 98.5% (95% CI: 97.8–99.0%) (Table 2). At the observed SARS-CoV-2 prevalence (24.5%) the positive predictive value (PPV) was 90.8% (95% CI: 86.8–93.9%) and the negative predictive value (NPV) was 85.0% (95% CI: 83.4–86.6%) (Table 1; S1 and S2 Figs). The overall agreement between the Ag RDT and rRT-PCR results was moderate (*k* = 0.5,), substantial (*k = 0*.*7*) in those with symptoms, and fair (*k = 0*.*4*) among those without. The performance of the Panbio^™^ Ag RDT is summarized in Table 2.

**Table 2:** The performance of the Panbio^™^ Ag RDT by clinical status, and Ct value.

	n	Ag RDT +ve	rRT-PCR +ve	Ag RDT -ve	rRT-PCR -ve	Sensitivity % (95% CI)	Specificity % (95% CI)	PPV % (95% CI)	NPV % (95% CI)	Kappa k (95% CI)
**Cases**										
All	2,245	257	551	1,668	1,694	46.6 (42.4–50.9)	98.5 (97.8–99.0)	90.8 (86.8–93.9)	85.0 (83.4–86.6)	0.5 (0.5–0.6)
Symptomatic	876	154	254	610	622	60.6 (54.3–66.7)	98.1 (96.7–99.0)	92.8 (87.7–96.2)	85.9 (83.1–88.4)	0.7 (0.6–0.7)
Asymptomatic	1,369	103	297	1,058	1,072	34.7 (29.3–40.4)	98.7 (97.8–99.3)	88.0 (80.7–93.3)	84.5 (82.4–86.5)	0.4 (0.4–0.5)
**Days post-onset of symptom**										
<5 days	594	106	158	428	436	67.1 (59.2–74.3)	98.2 (96.4–99.2)	93.0 (86.6–96.9)	89.2 (86.0–91.8)	0.7 (0.6–0.8)
*<3 days*	*363*	64	103	257	260	62.1 (52.0–71.5)	98.8 (96.7–99.8)	95.5 (87.5–99.1)	86.8 (82.4–90.5)	0.7 (0.6–0.8)
* 3-<5 days*	*231*	42	55	171	176	76.4 (63.0–86.8)	97.2 (93.5–99.1)	89.4 (76.9–96.5)	92.9 (88.2–96.2)	0.8 (0.7–0.9)
5–7 days	125	16	36	87	89	44.4 (27.9–61.9)	97.8 (92.1–99.7)	88.9 (65.3–98.6)	81.3 (72.6–88.2)	0.5 (0.3–0.7)
>7–14 days	157	32	60	95	97	53.3 (40.0–66.3)	97.9 (92.7–99.7)	94.1 (80.3–99.3)	77.2 (68.8–84.3)	0.6 (0.4–0.7)
≤7 days	805	146	237	557	568	61.6 (55.1–67.8)	98.1 (96.6–99.0)	93.0 (87.8–96.5)	86.0 (83.0–88.5)	0.7 (0.6–0.7)
>7–14 days	71	8	17	53	54	47.1 (23.0–72.2)	98.1 (90.1–100.0)	88.9 (51.8–99.7)	85.5 (74.2–93.1)	0.5 (0.3–0.8)
**Ct value** ≤**30**										
All	273	222	273	-	-	81.3 (72.2–91.6)	-	-	-	-
Symptomatic	162	141	162	-	-	87.0 (80.9–91.8)	-	-	-	-
Asymptomatic	111	81	111	-	-	73.0 (60.6–87.9)	-	-	-	-
**Ct value >30 - <40**										
All	278	35	278	-	-	12.6 (11.2–14.2)	-	-	-	-
Symptomatic	92	13	92	-	-	14.1 (7.7–23.0)	-	-	-	-
Asymptomatic	186	22	186	-	-	11.8 (10.2–13.7)	-	-	-	-

Ag RDT, antigen rapid diagnostic test; CI, confidence interval; Ct, cycle threshold; n, number of; NPV, negative predictive value; PPV, positive predictive value; rRT-PCR–real-time reverse transcriptase polymerase chain reaction.

In those with symptoms, false-negative results (Ag RDT-ve/rRT-PCR+ve) occurred in 100 (39.4%), and false positives(Ag RDT+ve/rRT-PCR-ve) occured in two percent (12/632) participants. Among the asymptomatic individuals, 65.3% (n = 194) had a false negative Ag RDT result, and 1.3% (n = 14) had a false positive result by Ag RDT.

Among the 876 symptomatic participants, the sensitivity was 60.6% (95% CI: 54.3–66.7%) (Table 2). In those with onset of symptoms <5 days sensitivity was 67.1%, declining to 53.3% among those with onset >7 days. Sensitivity in asymptomatic participants,was significantly lower at 34.7% (95% CI: 29.3–40.4%) compared to symptomatic ones (*p* <0.001). Specificity remained high in symptomatic (98.1%, 95% CI: 96.7–99.0%) and asymptomatic individuals (98.7%, 95% CI: 96.7–99.0%) (S4 Fig).

Regardless of the presence or absence of symptoms, the overall sensitivity of the Ag RDT test in 273 specimens with Ct values ≤ 30 was 81.3% (95% CI: 72.2–91.6). This reduced to 12.6% (95% CI: 11.2–14.2%) among the 278 individuals with Ct >30 (Table 2).

### Evaluation of the user’s experience of the Panbio^™^ Ag RDT

A total of 17 surveillance officers comprising 10 (59%) clinicians and 7 (41%) laboratory staff completed the survey. All 17(100%) surveillance officers found the test procedure easy to perform, and the manufacturers’ instructions were found to be clear and easy to follow. Four (23.5%) of the surveillance officers noted the difficulty in dispensing a sample onto the test device from the extraction tube when nasopharyngeal specimens were collected from individuals with thick mucus.

## Discussion

We present findings of a field evaluation of the Panbio^™^ Ag RDT conducted among symptomatic patients with onset of symptoms up to 14 days and asymptomatic individuals who had contact with individuals with confirmed SARS-CoV-2 infection within two days before detection of the index case and up to 14 days after exposure. Our evaluation was conducted in the third COVID-19 wave in Kenya, however, the majority of our participants were enrolled towards the end of the wave [13,14] where the median national prevalence was 9.6%(IQR 7.9–13.1%) [15]. The proportion of symptomatic individuals enrolled (54%) was higher than observed in the Kenyan population [13].

The overall sensitivity was low (46.6%), detecting slightly less than half of the rRT-PCR confirmed cases, therefore, missing more than half and classifying them as not having SARS-CoV-2 infection when they were indeed infected. In our evaluation, sensitivity varied with the presence or absence of symptoms, days since onset of onset, and viral load. As expected, sensitivity was higher among symptomatic individuals compared to asymptomatic ones (60.6% vs. 34.7%), and those with higher viral loads compared to those with lower ones (Ct value ≤30 vs. >30). Sensitivity increased with more recent onset of infection, being higher in those with onset of symptoms less than 7 days compared to those with onset >7 days (61.6% vs. 47.1%). By duration since onset of infection, sensitivity was highest among those with onset between 3 and 5 days. Symptomatic individuals had significantly higher viral loads (Fig 2), and with the highest sensitivity (87.0%) observed where Ct values ≤30, exceeding the WHO recommendation.

The low overall sensitivity observed may be due to the high proportion (61.1%) of asymptomatic participants, who had significantly higher Ct values and therefore more likely to test negative by Ag RDT. Antigen RDTs perform better in individuals with higher viral loads [7]. Sensitivity was lower than indicated in the manufacturer’s product insert (61.6% vs. 91.1%) [11] and the WHO minimum recommendation (80%) [8]. This is likely due to the selection of specimens with high viral loads by the manufacturer’s study for approval as hypothesized by *Albert et al*., *2021* in an evaluation where SARS-CoV-2 cultures were conducted [4].

The overall sensitivity was lower than reported in similar studies conducted in a multicenter study in Spain conducted among individuals of any age (57.3%, 95%CI: 48.3–95.8%) [16], Spanish adults in primary care centers (71.4%, 95% CI: 63.1–78.7%) [17], among Spanish individuals of any age (73.3%, 95% CI: 62.2–83.8%) [18], and those presenting within 7 days of onset or history of exposure (90.5%, 95% CI 87.5–93.6%) [19]. The lower sensitivity we observed could have been due to the relatively younger particpants (median age 31 years) enrolled in our evaluation compared to other studies with higher median age (39–51.5 years) [18,20]. A recent study demonstrated increasing viral load with increasing age [21] and resultant higher sensitivities. In addition, while we did not ascertain the variants in the specimens tested, the alpha variant and delta variants predominated the third wave in Kenya [22,23]. While some literature indicates that variants of concern (17,18) do not reduce the sensitivity and specificity of Ag RDTs, a recent study reported that sensitivity of the Panbio^™^ Ag RDT declined significantly in individuals infected with the alpha variant (53.0%) compared to those with non-alpha variants (89.0%) even after adjusting for viral load (p <0.002) [24] as was also demonstrated by *Wertenauer et*. *al* in the COVAG study [25]. Mutations occurring in the N protein of the Alpha variant may not be detected by the Panbio^™^ Ag RDT [26]. Laboratory studies have demonstrated a tenfold reduction in detection of the alpha variant by the Panbio^™^ Ag RDT [27]. Early laboratory data also demonstrated reduced sensitivity of other Ag RDTs in laboratory studies [28–30], while others have demonstrated no impact on performance [31]. Further clinical evaluations may be required to determine the impact of viral mutations on the performance on Ag RDTs.

The sensitivity observed among symptomatic individuals was similar to that observed in symptomatic Mexican adults (58.1%, 95% CI: 54.9–61.3%) [32]. However, it was higher than reported in a multicenter evaluation conducted among symptomatic children (60.6% vs. 45.4%) with the onset of symptoms within five days [20].

Sensitivity was higher among symptomatic individuals compared to asymptomatic ones. Similar findings were observed in other evaluations with higher sensitivity among symptomatic individuals compared to asymptomatic in a study in Mexico (58.1% vs. 26.3%) [32], in Spain (80.4% vs. 56.6%) [17], among Greek children (95.2% vs. 22.2%) [33], and among Swiss children (73% vs. 43%) [34]. However, a German study found similar sensitivities between the two groups (86.8% vs. 85.7%) [35]. The symptomatic individuals we enrolled in our evaluation had significantly lower Ct values than asymptomatic participants (Fig 2). Our evaluation further observed that irrespective of symptoms sensitivity was higher among those with Ct value ≤30 as reported by other authors [17,35–37]. We also observed significantly higher viral loads among symptomatic individuals compared to asymptomatic individuals (p <0.001) as has reported in recent literature [38,39]. In contrast, some studies found no difference in viral load between the two groups [40,41] while others found higher viral load among asymptomatic individuals [42,43]. Higher sensitivity has been observed among individuals presenting in the early symptomatic phase of their infection [19,32,35–37,42,44,45].

While rRT-PCR testing for SARS-CoV-2 is the reference standard for diagnosis, its limited access in our setting and provision of results in a clinically relevant timely manner to inform case management limits its clinical utility. Therefore, given reasonable sensitivity, individuals with a negative Ag RDT test in an individual meeting the clinical and epidemiological criteria may require a confirmatory test by rRT-PCR. The variation in sensitivity by presence or absence of symptoms, symptom duration, and viral load indicates that the kit may only be useful among individuals with high viral loads, especially those with symptoms.

The PPV (90.8%) observed at the prevalence of SARS-CoV-2 infection of 24.5% by rRT-PCR was similar to the projected PPV (89.6%.) assuming the minimum WHO recommended sensitivity (80%) and specificity (97.0%). The NPV (85.0%) was lower compared to that computed NPV (93.7%) assuming the minimum recommendations. Among individuals who tested negative by Ag RDT, the likelihood of being wrongly classified as uninfected was 15%. In 2021, the median positivity rate in Kenya was 7.4% (IQR, 3.2–13.1%) [46]. At the observed sensitivity and specificity and the national positivity rate, PPV would range between 50.2% and 82.8%, and the NPV 92.4%–98.2%).

The overall specificity remained high (98.5%) and was above the WHO recommendation [47] for both symptomatic and asymptomatic individuals and across the other subgroups (Table 2) as has been reported in other evaluations [32,35,36,44,48–51]. The Ag RDT had high specificity with less than (1.5%) of the tests (n = 26) giving a false-positive result. This small proportion represents individuals who would be falsely classified as infected and unnecessarily be managed as COVID-19 cases or self-isolated and their contacts traced and quarantined.

However, we observed over half (53%) of the Ag RDT results gave false-negative results with a higher proportion (65%) among the asymptomatic individuals. This observation emphasizes the recommendation by WHO to re-test symptomatic patients with rRT-PCR when they receive a negative Ag RDT result, especially in settings where SARS-CoV-2 prevalence is ≤ 5%. Further, as a screening tool for testing contacts of confirmed cases, the 65% false negatives among asymptomatic contacts represent the proportion that would falsely be classified as virus-free, not self-isolate, and potentially infect others.

The Panbio^™^ Ag RDT was found to be easy to use by the majority of the users with a quick TAT for clinical decision-making and implementation of preventive measures to contain transmission as observed in the literature [35,36]. The main challenge we observed in conducting Ag RDT testing using the Panbio^™^ Ag RDT kits was dispensing the specimen onto the test device in samples collected from individuals with thick mucus. This was, however, solved by ensuring that individuals blew their noses before specimen collection.

Our field evaluation had several strengths. The evaluation was conducted in the real-world setting across multiple sites under the point-of-care conditions which had a large sample size comprising two groups (symptomatic and asymptomatic individuals) thereby representing individuals at any point of the COVID-19 disease spectrum.

### Limitation

The number of persons enrolled in the two groups, symptomatic and asymptomatic, may not reflect the true prevalence in the general population as we evaluated the Ag RDT during a period of high COVID-19 prevalence. While rRT-PCR was used as the reference standard, it is not a gold standard as it may not truly differentiate replicating virus and residual RNA from recent infection among those with high Ct values. Residual RNA may not be viable and therefore not infectious [52].

## Conclusion

The overall sensitivity and PPV of the Panbio^™^ Ag RDT were much lower than expected. Sensitivity was acceptable in symptomatic individuals with Ct value ≤30. The specificity of the Ag RDT was high and satisfactory; therefore, a positive result may not require confirmation by rRT-PCR. The kit may be useful as a rapid screening tool for only symptomatic patients in high-risk settings with limited access to rRT-PCR. A negative result should be interpreted based on clinical and epidemiological information and may require retesting by rRT-PCR.

## Supporting information

S1 Fig The positive predictive value of the Panbio^™^ Ag RDT by clinical status and days post onset of symptoms, Kenya 2021. (TIF)

S2 Fig The negative predictive value of the Panbio^™^ Ag RDT by clinical status and days post onset of symptoms, Kenya 2021. (TIF)

S3 Fig The sensitivity of the Panbio^™^ Ag RDT by clinical status, and Ct values. (TIF)

S4 Fig The specificity of the Panbio^™^ Ag RDT by clinical status and days post onset of symptoms, Kenya 2021. (TIF)
