# Supplementary figures and images for: Diagnostic accuracy of the Panbio COVID-19 antigen rapid test device for SARS-CoV-2 detection in Kenya, 2021: A field evaluation
Source: PLoS One. 2023 Jan 25;18(1):e0277657. doi: 10.1371/journal.pone.0277657 (PMC9876661; doi:10.1371/journal.pone.0277657)

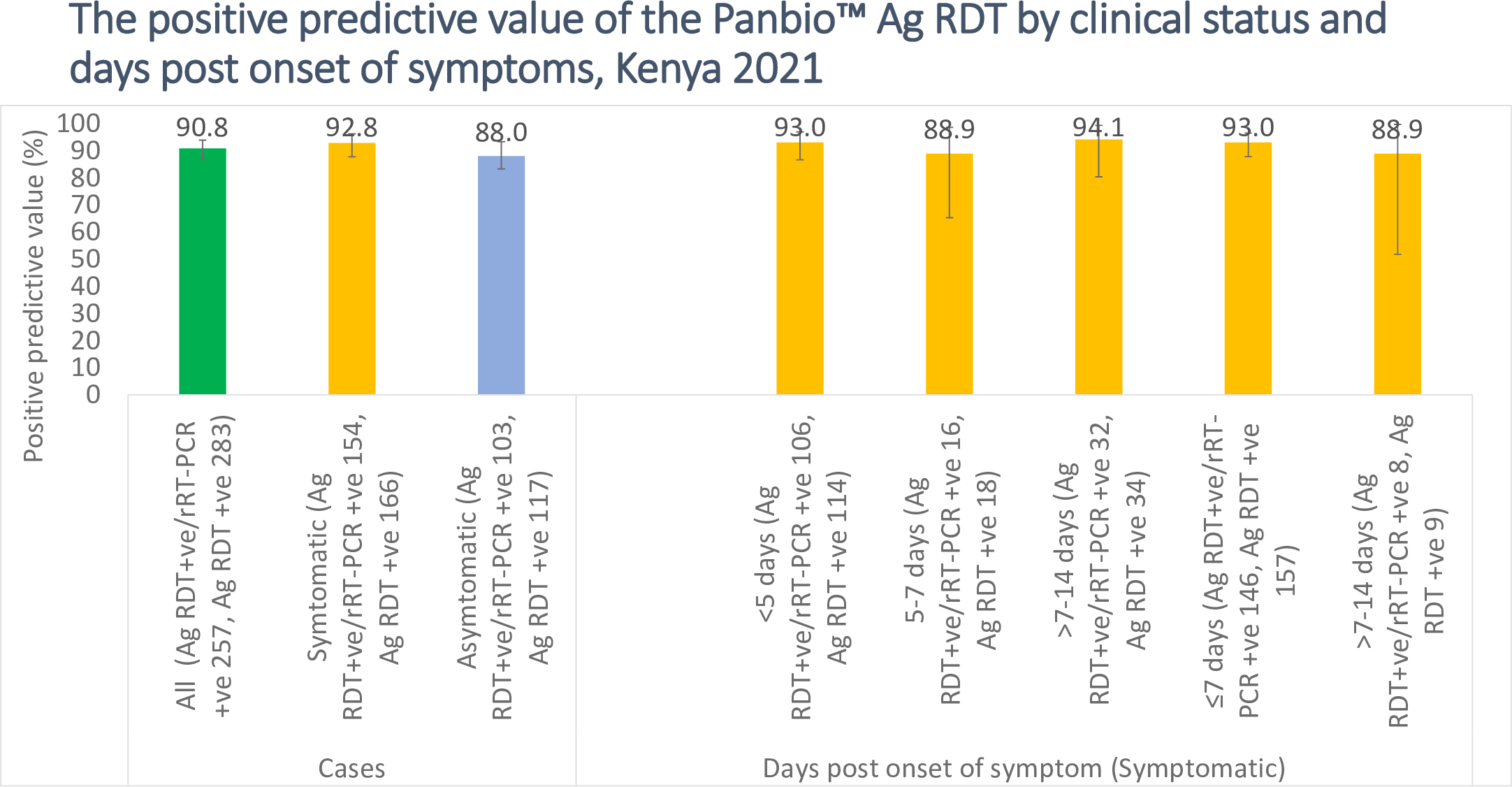

Supplement: S1 Fig — (TIF) [file pone.0277657.s001.tif]

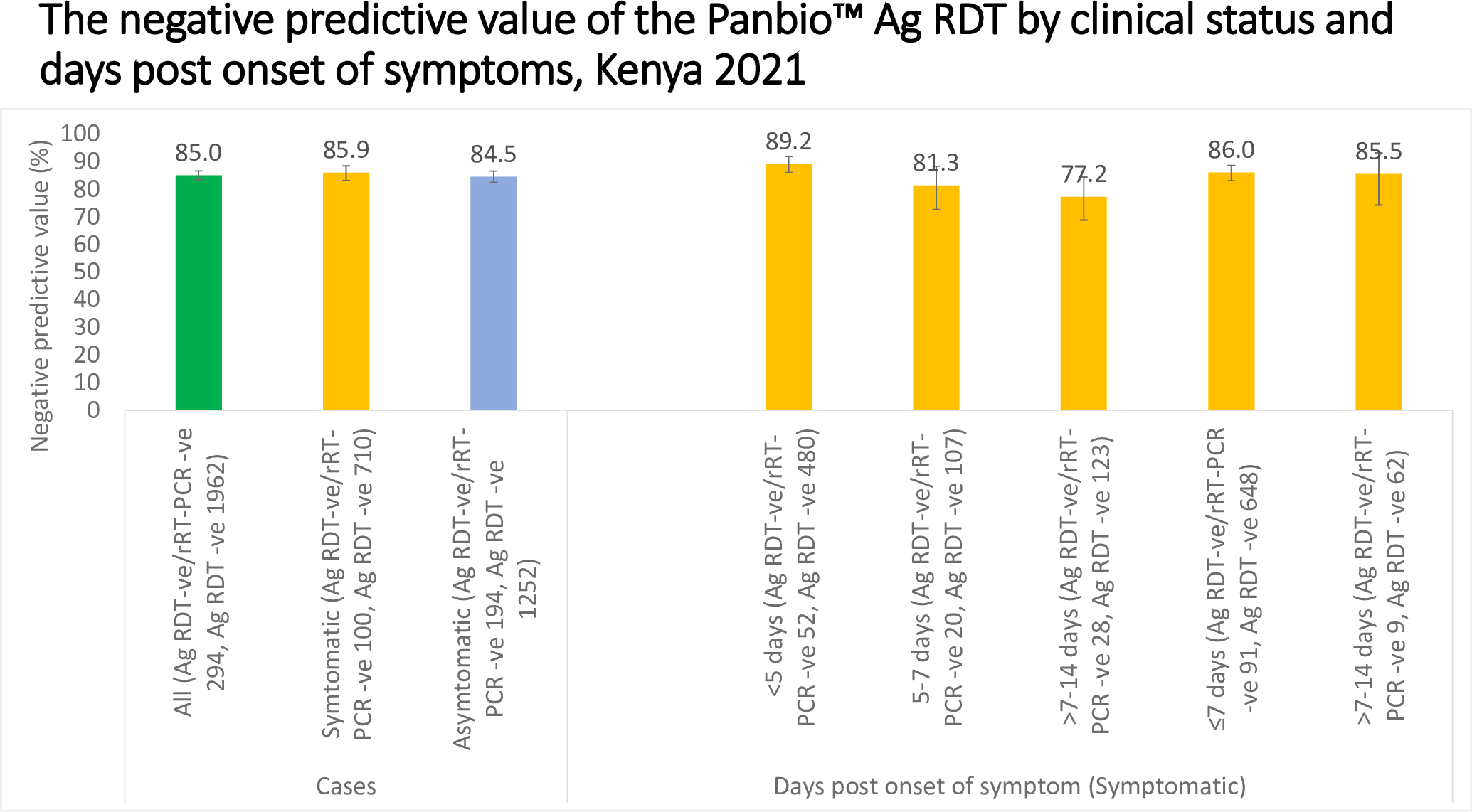

Supplement: S2 Fig — (TIF) [file pone.0277657.s002.tif]

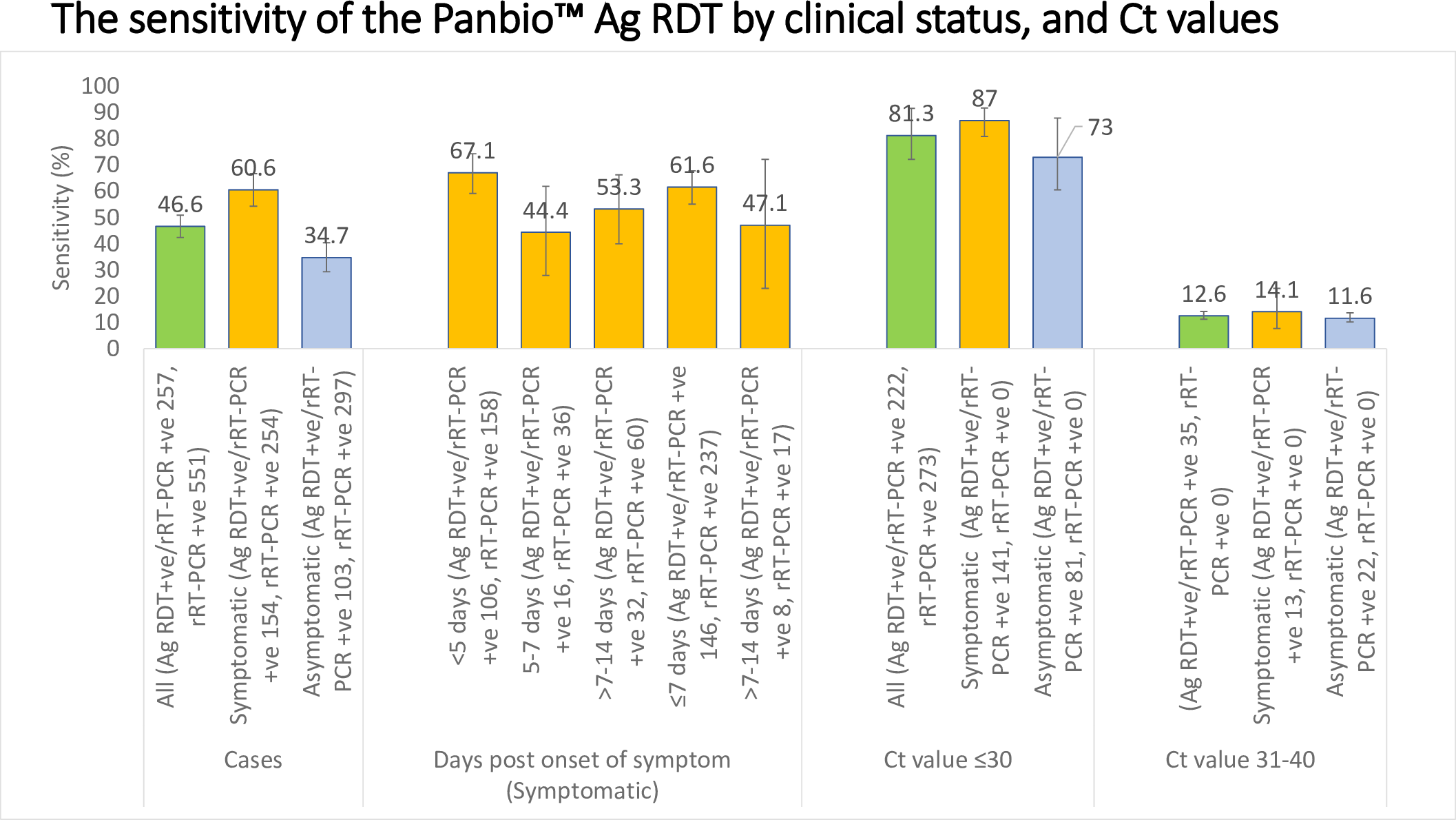

Supplement: S3 Fig — (TIF) [file pone.0277657.s003.tif]

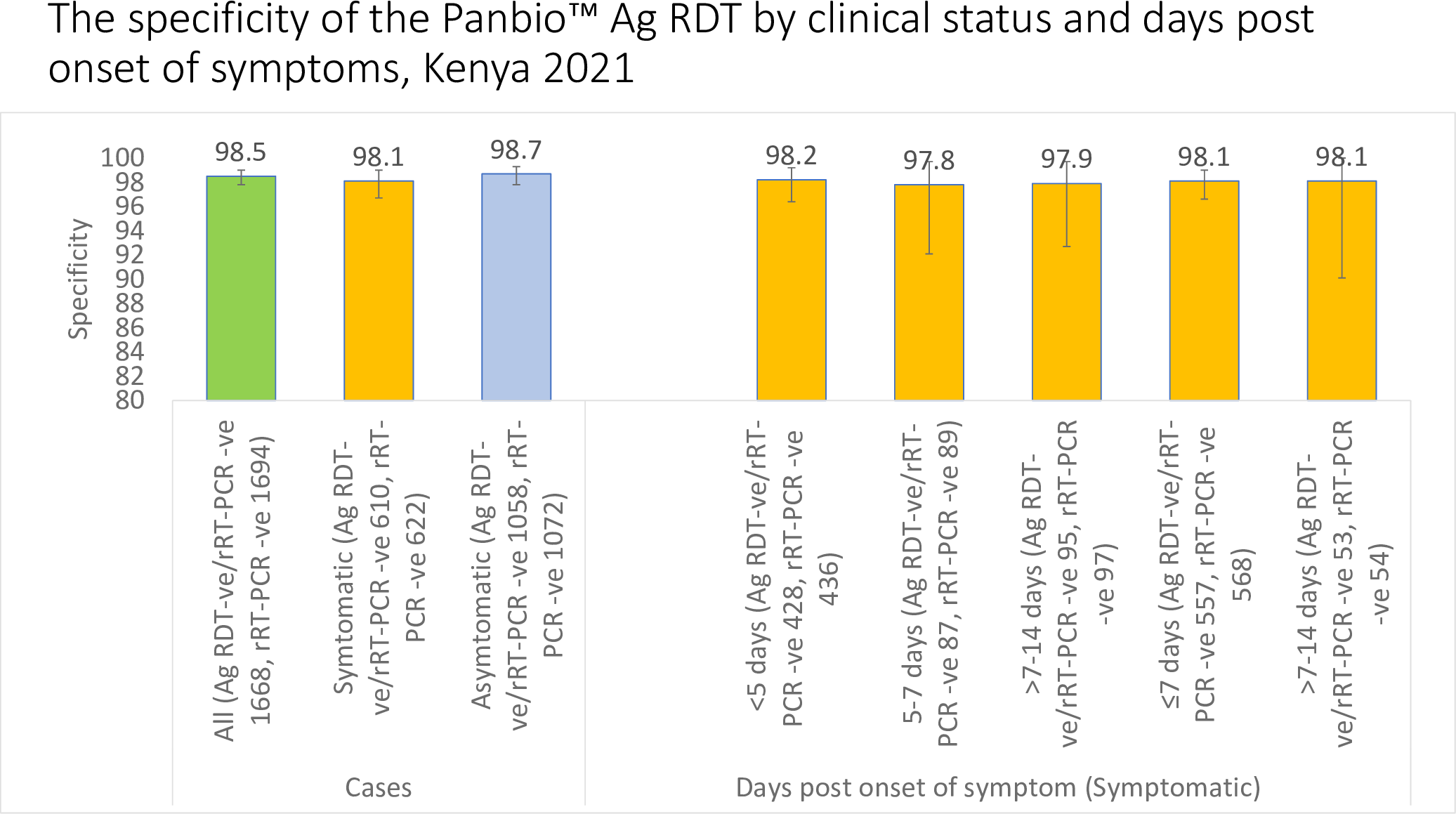

Supplement: S4 Fig — (TIF) [file pone.0277657.s004.tif]
